# Physical Activity and Education in the Care of IBD: The Role of the Physiotherapist—A Narrative Review

**DOI:** 10.3390/jcm14238602

**Published:** 2025-12-04

**Authors:** Zita Kovács, Péter Bacsur, Blanka Bernadett Kasza, Tamás Molnár, Andrea Domján

**Affiliations:** 1 Department of Physiotherapy, Faculty of Health Sciences and Social Studies, University of Szeged, 6726 Szeged, Hungary; 2 Department of Medicine, Albert Szent-Györgyi Medical School, University of Szeged, 6720 Szeged, Hungary

**Keywords:** physiotherapist, inflammatory bowel disease, fatigue, physiotherapy, exercise

## Abstract

Inflammatory bowel diseases (IBD), including Crohn’s disease (CD) and ulcerative colitis (UC), are chronic, immune-mediated conditions that significantly affect quality of life (QoL). The disease can cause extraintestinal manifestations, the most common of which is musculoskeletal involvement, which can lead to reduced physical activity (PA) and further impair QoL. In this narrative review, the literature was studied regarding the effects of PA types and patient education in IBD. There is growing evidence that regular PA and an active lifestyle have a positive impact on patients’ QoL, reduce symptoms, and contribute to maintaining remission. Aerobic and resistance training programs, when properly dosed, have been shown to be safe, improve physical condition, and have an impact on psychological well-being, while not increasing disease activity. On the other hand, there is no consensus on the safety of high-intensity training, so individualized, gradual training programs are recommended. The lack of PA and low levels of PA among IBD patients are partly due to fatigue, fear of symptoms, and joint pain, which may be caused by a lack of adequate education. A multidisciplinary approach and the involvement of physiotherapists are often lacking. Available data show that structured, patient-centered education programs and personalized exercise therapies can help increase PA and improve QoL. Overall, regular PA should be an important therapeutic adjunct to IBD treatment, but further research is needed to investigate training programs of appropriate intensity and frequency that can be used safely, and we also recommend assessing the need for patient education.

## 1. Introduction

Inflammatory bowel disease (IBD) is a chronic, immune-mediated disorder that primarily affects the gastrointestinal tract. The two primary forms are Crohn’s disease (CD) and ulcerative colitis (UC), both characterized by immune system dysregulation, alterations in the intestinal microbiota, and abnormalities in intestinal permeability [1]. In genetically predisposed individuals, chronic inflammation develops as a result of an abnormal immune response to altered gut flora, leading to alternating periods of disease activity and remission [2]. The global prevalence of IBD is estimated at approximately 3.9 million females and 3.0 million males, with an increasing trend observed in both developed and developing countries [3].

IBD can markedly impair patients’ quality of life (QoL), presenting with a wide range of gastrointestinal symptoms, including abdominal pain, cramping, diarrhea, and bloody stools. In addition, up to half of patients experience extraintestinal manifestations (EIMs), which further compromise QoL and complicate disease management [4]. In both CD and UC, the musculoskeletal system represents the most common site of extraintestinal involvement, with peripheral or axial arthritis and enthesitis occurring in approximately 5–25% of patients [5]. Joint pain and fatigue due to musculoskeletal involvement may lead to reduced physical activity (PA), which in turn contributes to decreased muscle strength and QoL, further exacerbating fatigue and diminishing patients’ ability to cope with stress [6].

In contrast, the current literature shows that regular exercise and an active lifestyle help to improve QoL of these patients, objectively reduce their symptoms and disease activity, and maintain remission [7,8]. It should be noted that malabsorption and pharmacological treatments for IBD are associated with an increased risk of osteoporosis, against which an active lifestyle may play an important preventive role [9].

Physiotherapists play a crucial role in the modern, multidisciplinary management of IBD, providing education on active living and ensuring appropriate mobility in both inpatient and outpatient settings during flare-ups. Despite this, research shows that the work of physiotherapists in the care of IBD patients is underutilized, often not even involving them in the multidisciplinary care of IBD patients [10,11]. Furthermore, there are currently no established, up-to-date guidelines on specialized physiotherapy approaches for patients with IBD, especially those with articular involvement.

Therefore, we aimed to review the literature on exercise recommendations for patients with IBD and examine the roles of education and physiotherapy in promoting PA.

## 2. Materials and Methods

In this narrative review, available literature was studied regarding the effects of PA types and patient education in IBD. To date, a systematic review has not been conducted due to the lack of robust data on patient education; therefore, we performed a narrative review of the available literature. The key focus of this review was on the evidence on PAs that can be undertaken by IBD patients, their effectiveness, and the need for patient education.

We searched the following electronic databases between 1 July and 31 August 2025, without language restrictions: PubMed (www.pubmed.ncbi.nlm.nih.gov), Google Scholar (https://scholar.google.com), ScienceDirect (www.sciencedirect.com), and SZTE Klebersberg Library and Archives (www.ek.szte.hu). The following search key was entered into each database: inflammatory bowel disease OR Crohn’s disease OR ulcerative colitis AND exercise OR physical activity OR training OR patient education. Available English full-text articles were included regardless of their year of publication, including original studies, reviews, and guidelines. Animal studies, non-English publications, publications unrelated to the topic, pediatric research, and duplicate records were excluded. Additionally, abstracts and conference proceedings were not considered. The narrative literature review was organized and presented according to pertinent thematic areas relevant to the research focus. When selecting, we took into account that the review wanted to emphasize the role of physiotherapists, so we included articles that contained specific details about PA, whether it was intensity or type.

**Figure 1:**
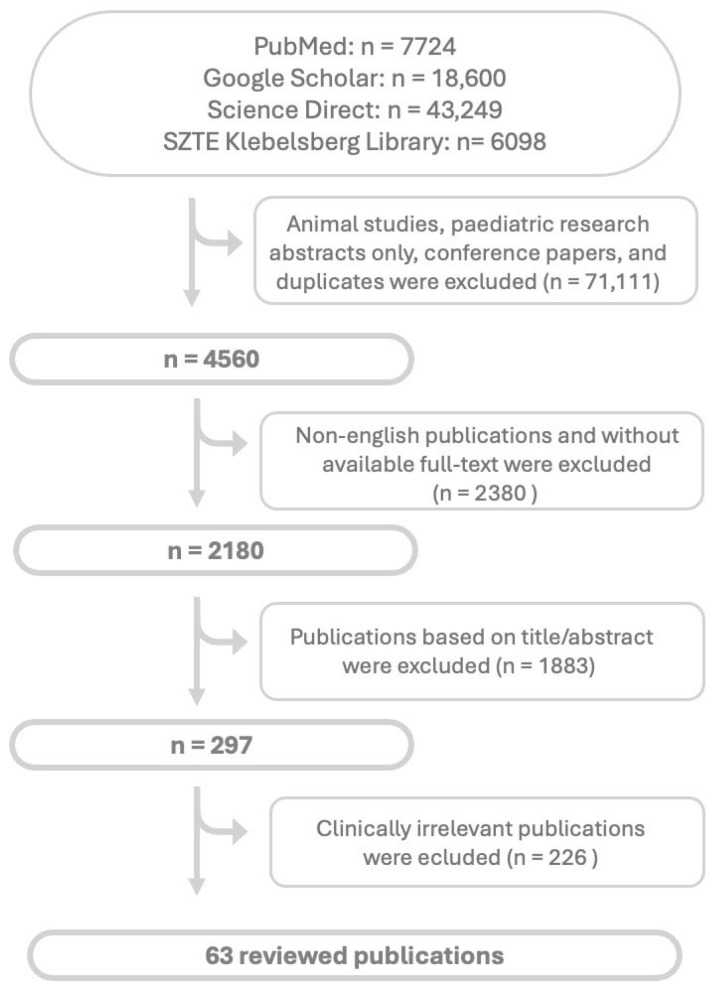
Flowchart of article search and selection. Abbreviations: *n*: nubmer of articles.

As this was a narrative literature review, no statistical analyses were conducted. Possible limitations were described in further section. Ethical approval was not required, as the study did not involve primary research with human participants. Flowchart of searched and selected articles are shown in Figure 1.

## 3. Narrative Review

### 3.1. Role of Physical Activity in IBD

Lower levels of PA have been consistently observed in patients with IBD compared to healthy controls; however, the role of exercise as a therapeutic intervention remains to be fully established [12,13,14]. The physical dimension of fatigue encompasses both the subjective experience of reduced strength and the objectively measurable decline in PA or functional capacity [15]. Musculoskeletal manifestations represent the most common extraintestinal complication of IBD, affecting up to 40% of patients [5,16]. Joint pain and fear of physical exertion contribute to decreased muscle strength and functional capacity in this population [16]. Approximately 30% of patients with IBD believe that PA may trigger disease exacerbations, which likely contributes to reduced exercise levels. Notably, many individuals with IBD perceive their condition as a significant barrier to engaging in regular PA, due in part to symptoms such as abdominal discomfort and urgency, but predominantly as a result of persistent fatigue. This fatigue restricts exercise tolerance, creating a self-perpetuating cycle of physical inactivity, declining fitness, and worsening exhaustion [17,18,19].

Fortunately, PA is increasingly recognized as a key factor in promoting overall health, psychosocial well-being, and QoL in individuals living with IBD [20]. Regular exercise has been shown to reduce stress, enhance QoL, improve bone density, and decrease the risk of colorectal cancer generally [21]. Although the precise physiological mechanisms remain unclear, it is hypothesized that PA induces the release of anti-inflammatory cytokines. Furthermore, studies have demonstrated a significant reduction in C-reactive protein (CRP) levels among IBD patients who engage in regular PA. Additionally, exercise plays a critical role in the prevention and management of obesity, an increasing concern within this patient population [22,23,24].

### 3.2. Recommended Forms of Exercise in IBD

PA is recommended not only for its general health benefits but also for its potential to alleviate IBD-related complications by enhancing immune function, supporting mental well-being, improving nutritional balance, preserving bone mineral density, and counteracting the loss of muscle mass and strength. Additional benefits include improved joint flexibility and reduced joint pain [12,25,26]. Furthermore, Koutouratsas et al. reported that exercise produced favorable changes in the gut microbiome [27].

Guidelines developed specifically for patients with IBD in 1998 recommended PA to promote general health, counteract muscle wasting, and improve bone density [28]. These guidelines advised 20–60 min of aerobic exercise two to five times per week, complemented by resistance training at least twice weekly. According to several physical exercise interventions carried out with people suffering from IBD, walking is a safe, practical and easy-to-do form of aerobic exercise which should be recommended. Although, general recommendations regarding the optimal form of aerobic exercise are currently not available, existing data suggest that swimming, pedaling on a recumbent bike, or weight-bearing walking may be advisable for IBD patients [29]. This review suggests that patients with CD should be offered two primary forms of physical intervention: aerobic exercise and resistance training [29].

Programs incorporating both aerobic and resistance training have been shown to improve physical fitness, achieve high compliance rates, and demonstrate a favorable safety profile without significant worsening of disease activity [30,31,32]. Both high- and moderate-intensity interval training appear to be safe for patients with IBD, while walking interventions have also been associated with improvements in QoL [33,34,35,36,37]. Other investigators have reported that moderate-intensity exercise, such as walking or yoga, led to significant improvements in QoL among patients with inactive or mildly active CD [38,39,40,41,42].

The available clinical data and opinions are not consistent regarding high-intensity training. Some studies warn that acute, very strenuous, or forced exertion may trigger the release of pro-inflammatory cytokines [12], which can temporarily worsen inflammatory conditions, as acute inflammatory profiles may develop after such workouts, while others have reported positive effects.

Nieman et al. (2012) examined the effect of high-intensity, prolonged cycling on acute inflammatory responses and found an increase in white blood cell count following the training sessions [43]. Sanchez et al. showed that intense physical exercise induces gastrointestinal changes that are relatively rare during low-intensity activity, including diarrhea, gastrointestinal bleeding, abdominal pain, and ischemic colitis [44]. In a small prospective cohort study of patients with IBD engaging in high-intensity exercise, 2 out of 10 participants experienced symptom exacerbation after exercise [45]. In the study by Bilski et al., intense training was shown to increase systemic inflammation and cytokine levels, leading to the worsening of gastrointestinal symptoms [46]. Furthermore, Al-Nimer et al. demonstrated that moderate-intensity exercise may be more effective than high-intensity exercise in stimulating the release of myokines [47].

The opposing perspective, however, evaluates intense PA in a positive light, suggesting that high-intensity training might also be feasible for patients with IBD. Keohane et al. found that it had a positive effect on gut microbial parameters, including species diversity, microbial functionality, and the upregulation of metabolic potential [48].

Taylor et al. and Buffart et al. demonstrated that higher-intensity exercise effectively improved the physical functioning aspects of QoL in colorectal cancer patients (physical performance, pain, limitations), suggesting that IBD patients may also experience similar benefits from such interventions through interactions between various gastrointestinal and disease-related factors [49]. Recently, DuBois and colleagues conducted a survey amongst 2052 UC patients, recording disease-related outcomes and levels of PA. The results showed that compared to moderate or low-intensity exercise, strenuous PA was associated with greater benefits in UC-related health outcomes, including disease activity index, sleep, anxiety, and fatigue [50].

In patients with severe disease activity, low-intensity exercises—such as bed-based movements, resistance band routines, and hand grip exercises—are recommended to help maintain baseline physical function. For those with moderately active disease, a structured endurance training program is advisable. Aerobic exercise should be performed at approximately 60% of the individual’s maximum heart rate, while resistance training may commence at 60% of the one-repetition maximum. Both modalities should follow a gradual progression model, with intensity increased by approximately 10–20% over time to promote safety and adaptation [47,51]. Patients are advised to consult a physician before initiating a regular exercise program, as exercise tolerance may be reduced during active disease. Common IBD-specific barriers to PA include joint and abdominal pain, muscle weakness, health concerns, embarrassment, and limited toilet access; however, fatigue remains the most frequently reported limitation [52]. Although no definitive exercise guidelines currently exist for patients with IBD, experts provide a range of recommendations tailored to the active phase of disease. Further research is warranted to refine these recommendations and develop specific, evidence-based exercise guidelines for this population [53].

In summary, although perspectives on optimal exercise modalities may vary, there is a broad consensus that PA provides substantial benefits for individuals with IBD, provided that its intensity and frequency are appropriately tailored to the patient’s current disease activity. Accordingly, physiotherapist-led, individualized exercise programs may represent the most effective and efficient approach to promoting safe and sustainable PA in this population. Based on the included studies, it is important to highlight that differences in follow-up duration, sample size, disease activity, and intervention parameters may limit their comparability and potentially influence our results. Table 1 contains details of the studies on PA included in this review.

### 3.3. The Role of Physiotherapists in Regular IBD Care

In cases of fatigue and anxiety, patient education is of paramount importance, as physical activity itself can help reduce fatigue levels. Therefore, patients should be encouraged to stay active despite feeling tired, since their symptoms are likely to improve as a result [32]. Physiotherapists have a fundamental role to educate patients regarding their PA during IBD care.

Brevinge and colleagues found that maximal workload measured on an ergometer was inversely related to the extent of small bowel resection, which should be taken into consideration by physiotherapists when designing training programs. Accordingly, patients who have undergone extensive intestinal resections are advised to avoid high-energy-demanding PAs [54].

Progressive, low-intensity exercise is potentially an effective method for increasing bone density in CD and is therefore strongly recommended for patients. If performed regularly over time, the increase in bone mineral density (BMD) can help reduce the risk of osteoporotic fractures [25]. Physiotherapists can help to manage osteoporosis in a multidisciplinary approach.

During mild or moderately active phases of the disease, it is recommended that patients engage in at least 30 min of moderate-intensity endurance and resistance exercise per week [29]. Research shows that studies examining PA rarely include patients who are in relapse; therefore, low-intensity training currently appears to be the safest option for them. As studies are not consistent regarding high-intensity training, and in line with several authors’ recommendations, we also suggest conducting larger, prospective, multi-center studies involving patients with disease activity [52].

When selecting the type of training, it is always important to consider any comorbidities. Physiotherapists have a comprehensive understanding of the types of exercises suitable for conditions such as diabetes, hypertension, or low back pain, allowing them to tailor programs individually. For this reason, it would be beneficial to integrate physiotherapists into the multidisciplinary care team [55,56].

In addition to prescribing safe and effective exercise programs, physiotherapists play a key role in monitoring, motivating and ensuring long-term adherence to exercise programs. Their training allows them to assess physical capacity, movement patterns and functional limitations, allowing them to tailor the intensity and type of exercise to the patient’s current disease state and energy level. In chronic conditions such as IBD, where fatigue, pain, and fluctuating symptoms are common, individual supervision by a physiotherapist can help prevent overuse and the exacerbation of exercise-induced symptoms. In addition, physiotherapists can educate patients on postural correction and breathing exercises, all of which can contribute to improving QoL and reducing disease burden.

Integrating physiotherapists into IBD multidisciplinary teams not only increases the safety and effectiveness of exercise interventions, but also promotes a more holistic approach to care. Their collaboration with gastroenterologists, dietitians, psychologists, and nurses promotes comprehensive rehabilitation that addresses both the physical and psychosocial aspects of the disease. In the long term, such multidisciplinary collaboration can improve treatment adherence, reduce complications, and support the overall well-being of patients with IBD [55,56].

There is a lack of literature examining the effects of physiotherapist-guided therapy in patients with IBD; therefore, we strongly recommend conducting further research in this area.

### 3.4. The Role of Patient Education

Unfortunately, evidence from cross-sectional studies indicates that individuals with IBD generally engage in low levels of PA, a trend that is particularly evident among those with moderate to severe disease activity, in whom participation in regular exercise is markedly reduced [14,57]. In a study by Wang et al., most participants expressed unfavorable attitudes toward exercise, influenced by factors such as active disease symptoms, difficulties in symptom control, limited knowledge regarding appropriate PA, and low confidence in their ability to exercise effectively [57].

Negative perceptions about the value of PA during flare-ups were also reported. These attitudes were further compounded by insufficient understanding of exercise-related principles, including unfamiliarity with proper techniques and difficulty in determining appropriate intensity and modality. Moreover, variability in recommended exercise approaches and the infrequent provision of structured exercise prescriptions by healthcare professionals contributed to a lack of guidance and clarity regarding PA among many participants [57].

On the other hand, data suggest that prolonged physical inactivity or bed rest can lead to multiple complications, including disuse-related muscle atrophy, joint contractures, thromboembolic events, and the development of insulin resistance, which can delay recovery in patients experiencing more active phases of the disease [58,59,60]. Wang et al. emphasize the importance of developing evidence-based, practical exercise guidelines [57]. Such initiatives are essential to provide clinicians with a solid scientific foundation to enhance patient education and promote greater engagement in PA. Healthcare professionals play a pivotal role in this process by designing safe, individualized exercise programs, fostering patient motivation, and creating supportive environments that encourage exercise. In light of these findings, a comprehensive approach is recommended, including tighter control of disease activity, dissemination of disease-specific and exercise-related education via multiple platforms, development of detailed personalized training plans, and implementation of multifaceted social support systems to encourage and sustain patient participation in PA [57]. These patients require a multifaceted and patient-centered approach to their care [61].

Given the multifaceted impact of IBD on QoL, a multidisciplinary approach is essential—one that integrates comprehensive discussions regarding therapeutic options, potential surgical interventions, mental health considerations, and lifestyle modifications. Achieving optimal care outcomes requires sustained, clear, and empathetic communication between healthcare providers and patients, fostering active engagement and informed decision-making. Notably, low health literacy among individuals with IBD has been associated with increased psychological distress, heightened disease activity, and poorer overall health outcomes, including reduced health-related QoL [62,63].

Improving health literacy has the potential to enhance healthcare outcomes by strengthening patients’ self-efficacy, promoting the timely and appropriate use of healthcare services, and supporting engagement in preventive care [64]. In the context of IBD, individualized patient education has been shown to improve disease understanding, facilitate informed decision-making, and enhance adherence to medical treatments—factors that contribute to increased patient satisfaction and reductions in outpatient visits and overall healthcare costs [65]. Conversely, the absence of structured educational programs and reliable resources may lead patients to seek information from unregulated or biased sources. Therefore, implementing a standardized, evidence-based framework for patient education is essential to optimize disease management and empower individuals living with IBD [66].

On the other hand, studies have demonstrated that patients’ physical symptoms are significantly influenced by their psychological state. In particular, the interplay between emotions and joint pain has been confirmed by multiple investigations [67]. Zhang et al. showed that health education incorporating narrative medicine alongside an online patient support group improved the health status of individuals with IBD [62]. Consequently, this model warrants further development and broader implementation. Healthcare professionals should enhance their narrative and empathetic skills while actively facilitating the creation and maintenance of patient support groups [67,68].

Notably, professional guidance was widely recognized as a key factor in fostering a positive attitude towards exercise, with participants expressing a strong need for such support. Therefore, it is recommended that clear, evidence-based exercise guidelines for IBD be developed to ensure patients receive safe and practical advice to adopt a healthy lifestyle and maximize the benefits of PA [57].

We identified several studies in which educational materials were developed, and patient education was delivered; however, the multidisciplinary teams involved did not include physiotherapists [69]. For instance, in the educational materials described by Al-Ani et al., physiotherapists were notably absent from both the team and the development process [70].

It should be highlighted that several factors may influence the overall level of PA, such as life circumstances, family and financial situation, availability of training opportunities, workplace support, and comorbidities. We believe that the impact of some of these factors on PA can be modified through broad patient education. Simple, quick, and minimally equipment-dependent training options could be recommended, while employer support may play a crucial role in facilitating PA. With the support of professionals, patients can be helped to identify the barriers they face, and targeted interventions can be developed to address them. Growing up in a physically active family leads to higher levels of PA through learned behavioral patterns. Table 2 contains details of the studies on patient education included in this review.

## 4. Discussion

While recent systematic reviews [71,72,73] have addressed this topic, we provide a more comprehensive overview that highlights the importance of multidisciplinary team-based care and patient education, and further elucidates the role of physiotherapists in clinical practice from a narrative, general viewpoint. In this review, we aimed to explore the most appropriate and safe recommended PA forms for IBD patients; however, this task was challenging due to the varying numbers of subjects and inconsistent measurements in the studies, which may have limit the interpretation. We also integrated the recommended intensity and duration of regular PA for its effectiveness and provided a framework for its implementation in clinical practice. The topic is particularly relevant as PA plays an increasingly important role in the treatment of chronic diseases, including IBD. There were many randomized controlled trials (RCTs) on PA in IBD, but the form of training, population characteristics, and duration varied most of the time. The study extensively examines the literature, RCTs, narrative reviews, and recommendations related to PA and education for patients with IBD.

As the study was not a systematic review with meta-analysis, no statistical analysis was performed. This limits the generalizability of the results, and the subjectivity of the assessment cannot be ruled out. Furthermore, we were unable to control potential confounders amongst the included studies. The search was limited to English language results, which may have introduced language bias. Limitations of the published literature included variable sample sizes, differences in intervention components, and study designs, variable baseline IBD activity, and outcome measures. It would have been worthwhile to include randomized controlled trials that used intent-to-treat analysis or duration-specific exercise protocols.

Based on our review, we found that studies investigating high-intensity training in patients with IBD are limited. Conducting randomized controlled trials in this area would be highly valuable, as they would allow for a clearer understanding of both the positive and negative effects of high-intensity exercise. Further investigation into the relationship between PA and the gut microbiota is warranted, as emerging evidence suggests that their interaction may play a key role in metabolic health, immune function, and overall well-being. In addition, research on PA during disease flare-ups is scarce, despite the importance of identifying exercise modalities that can be safely recommended to patients experiencing relapses. Regarding patient education, we suggest implementing physiotherapist-led educational interventions, which could help clarify the potential limitations and safety considerations of PA. Furthermore, we recommend developing guidelines on the feasibility and role of a multidisciplinary team approach. Further future directions could include epidemiological or clinical trial data to understand better the IBD-specific outcomes that regular PA may influence (such as induction and maintenance of clinical remission, and effects on intestinal inflammation), as well as the most effective components and duration of PA needed to achieve desired outcomes.

## 5. Conclusions

In conclusion, assessing patients’ attitudes and beliefs about PA may help improve understanding of the factors contributing to low activity levels, and enhancing access to regular exercise could potentially support the integration of PA into therapeutic practice. This narrative review suggests that, while growing evidence indicates potential benefits of regular PA on symptom management, QoL, and disease activity in individuals with IBD, implementing exercise interventions remains challenging. Physical inactivity is often associated with fatigue, fear of symptom exacerbation, and joint pain, which might be alleviated through targeted patient education. The involvement of physiotherapists, the development of individualized exercise programs, and structured, evidence-informed educational interventions may represent promising approaches to enhance patients’ PA and overall well-being. Based on the current literature, moderate-intensity walking appears to be a feasible option for individuals with IBD, though recommendations should be interpreted with caution. Moreover, involving multidisciplinary professionals—including physicians, IBD nurses, dietitians, psychologists, and physiotherapists—may help ensure that patients receive coherent and comprehensive guidance. Future research is needed to establish clear, IBD-specific, evidence-based exercise recommendations, particularly those tailored to varying levels of disease activity and to cases with EIMs.

## Figures and Tables

**Table 1:** Details of included relevant observational and randomized trials regarding physical activities.

Author (s)	Type of Exercise/Questionnaire	Study Design	Disease	No. of Patients	Duration	Results
Ng et al. (2007) [35]	low intensity walking	RCT	CD	T = 32 IV = 16 C = 16 (remission to mild disease activity)	12 weeks (3/week)	Improved QoL with the intervention.
Loudon et al. (1999) [36]	walking program	prospective	CD	T/IV = 12 (remission to mild disease activity)	12 weeks (3/week)	All measures showed improvement by the study’s end, with a trend toward BMI reduction and no disease flares.
Lamers et al. (2021) [37]	walking program	prospective	UC + CD	T= 56 IBD = 37 C = 19 (disease activity was not defined)	4 days	Changes in cytokine concentrations were similar between IBD and non-IBD walkers.
Robinson et al. (1998) [25]	low-impact exercise program of increasing intensity	RCT	CD	T = 117 IV = 60 C = 57 (disease activity was not defined)	12 months	There was a statistically significant increase in BMD at the greater trochanter.
Sharma et al. (2015) [39]	Yoga	RCT	UC + CD	T = 100 Yoga: UC = 30 CD = 20, standard treatment: UC = 30 CD = 20 (disease in remission)	8 weeks (1 h/day)	There was a significant reduction in state anxiety.
Klare et al. (2015) [34]	moderate intensity running	RCT	UC + CD	T = 30 IV = 15 C = 15 (mild to moderate disease activity)	10 weeks	Scores on the IBDQ social subscale significantly improved.
Elsenbruch et al. (2005) [41]	stress management training, moderate exercise	RCT	UC	T = 45 IV = 15 C = 15 healthy = 10 (remission to mild disease activity)	10 weeks	Patients in the intervention group had significantly greater improvements on the SF-36 and IBDQ Bowel Symptoms scales compared to controls.
Jones et al. (2020) [30]	combined impact and resistance training	RCT	CD	T = 47 IV = 23 C = 24 (disease in remission)	6 months	Improved BMD and muscle function were observed.
Cronin et al. (2019) [31]	combined aerobic and resistance training (moderate intensity)	RCT	UC + CD	T= 20 IV = 13 C = 7 (disease in remission)	8 weeks	Physical fitness improved without worsening disease activity, along with changes in body composition.
van Erp et al. (2021) [32]	aerobic- and progressive-resistance training at personalized intensity	prospective	CD + UC/IBD-U	T = 25 UC = 3 CD = 21 IBD-U = 1 (disease in remission)	12 weeks	A personalized exercise program significantly improved fatigue, HRQoL, and cardiorespiratory fitness.
Seeger et al. (2020) [42]	endurance training or Muscle training	RCT	CD	T = 45 C = 13 IV = 32 (remission to mild disease activity)	12 weeks	Both forms of exercise improved strength and well-being, and increased QoL in intervention groups.
Tew et al. (2019) [33]	HIIT and MICT	RCT	CD	T = 36 (HIIT = 13 MICT = 12) C = 11 (remission to mild disease activity)	12 weeks	HIIT did not worsen disease activity, and was associated with few adverse events.
Gerbarg et al. (2015) [24]	BBMW (breathing, movement, meditation)	RCT	UC + CD	T= 29 IV = 16 C = 13 (disease activity was not defined)	26 weeks	The BBMW group showed significant improvements from baseline to week 6.
Ratajczak-Pawłowska et al. (2023) [26]	International Physical Activity Questionnaire	Cross-sectional	UC + CD	T = 50 C = 24 (disease activity was not defined)		BMD scores were significantly lower in IBD patients than in healthy controls, with no differences in PA duration among the groups.
Holik et al. (2019) [23]	Physical Activity Questionnaire	Cross-sectional	UC + CD	T = 312 (disease activity was not considered)		24% of patients were not on therapy, and daily PA was linked to inactive disease.

Table are ordered according to the type of the PA examined. Abbreviations: IBD: inflammatory bowel disease, UC: ulcerative colitis, CD: Crohn’s disease, T = total, IV = intervention, C = control, PA: physical activity, QoL: quality of life, HIIT: high-intensity interval training, MICT: moderate-intensity continuous training IL: interleukin, BBMW: breath-body -mind workshop, IBDQ: inflammatory bowel disease questionnaire, IPAQ: international physical activity questionnaire, TNF: tumor necrosis factor, BMI: body mass index, RCT: randomized-controlled study.

**Table 2:** Summary of trials included in the review on education.

Author (s)	Study Design	Method	Results
Tormey et al. (2019) [63]	prospective	interview with 112 patients	Low health literacy in IBD is linked to poorer perceived health, higher depression rates, and more active symptoms, particularly in Crohn’s disease.
Zare et al. (2020) [64]	cross-sectional	interview with 26 patients	Participants placed the greatest emphasis on self-care and psychological coping strategies.
Robinson et al. (2001) [65]	RCT	206 patients (The interval between relapse and treatment, and rates of primary and secondary care consultation, QoL, and acceptability to patients.)	Self-management in ulcerative colitis helps deliver treatment faster, reduces doctor visits, and does not increase health risks.
Mikocka-Walus et al. (2014) [69]	cross-sectional	online mixed-methods survey was conducted with health professionals caring for IBD patients	Among 135 respondents, 50% were GI physicians, 34% nurses, 8% psychologists, 4% dietitians, 2% surgeons, 1% psychiatrists, and 1% physiotherapists.

Abbreviations: IBD: inflammatory bowel disease, QoL: quality of life, RCT: randomized-controlled study, GI physicians: gastrointestinal physicians.

## Data Availability

AD is the guarantor of the article. Original data is not created. All authors have read and agreed to the submitted version of the manuscript. The current manuscript, including related data and figures, has not been previously published and is not under consideration elsewhere.

## Abbreviations

The following abbreviations are used in this manuscript:

IBD	Inflammatory bowel diseases
CD	Crohn’s disease
UC	ulcerative colitis
QoL	quality of life
EIMs	extraintestinal manifestations
PA	physical activity
CRP	C-reactive protein
